# Identification of ARV1 Gene Mutations in Three Pediatric Cases of Developmental and Epileptic Encephalopathy

**DOI:** 10.7759/cureus.82903

**Published:** 2025-04-24

**Authors:** Feras A. Majeed Buhusayen, Mohamed Alashraaf, Raafat Hamad Seroor H Jadah

**Affiliations:** 1 General Surgery, Bahrain Defence Force Hospital, Royal Medical Services, Riffa, BHR; 2 General Surgery, King Hamad University Hospital, Royal Medical Services, Riffa, BHR; 3 Pediatric Neurology, Bahrain Defence Force Hospital, Royal Medical Services, Riffa, BHR

**Keywords:** arv1 gene mutation, developmental and epileptic encephalopathy (dee38), infantile seizure onset, neurodevelopmental disorder, whole-exome sequencing (wes)

## Abstract

The ARV1 gene produces a protein made up of 271 amino acids that helps transport fats across membranes within the cell’s endoplasmic reticulum (ER), a key area involved in processing lipids. This protein is related to an enzyme called ACAT2, which is important for managing cholesterol and fat levels in the body. This protein features an N-terminal zinc-binding motif located in the cytosol, followed by multiple domains that span the ER membrane, and concludes with a C-terminus that terminates in the ER lumen. ARV1 deficiency clinically manifests as autosomal recessive developmental and epileptic encephalopathy 38 (DEE38) in humans.

In this report, we share three pediatric cases presenting with early-onset epileptic encephalopathy and significant developmental delay. Whole-exome sequencing (WES) identified two pathogenic ARV1 variants: p.Cys61Tyr (missense) and p.Phe144Argfs5* (frameshift), both predicted to severely disrupt protein structure and function. These findings add to what we know about how mutations in the ARV1 gene can lead to developmental and epileptic encephalopathy (DEE38), and they strengthen our understanding of the gene’s role in brain development. The children in our report also show how widely the symptoms of ARV1-related conditions can vary from case to case. Their experiences highlight just how important early genetic testing can be, especially for young patients with unexplained seizures and developmental challenges. Our report contributes to understanding the spectrum of complex neurological conditions. By sharing these cases, we’re adding to the growing knowledge about ARV1-related encephalopathies and reinforcing why this gene deserves a place in targeted epilepsy genetic panels.

## Introduction

The ARV1 gene produces a protein made up of 271 amino acids that helps transport fats across membranes within the cell's endoplasmic reticulum (ER), a key area involved in processing lipids. This protein is related to an enzyme called ACAT2, which is important for managing cholesterol and fat levels in the body [1]. This protein features an N-terminal zinc-binding motif located in the cytosol, followed by multiple domains that span the ER membrane, and concludes with a C-terminus that terminates in the ER lumen [1,2]. The ARV1 gene plays a significant role in maintaining homeostasis of cellular lipids by regulating the distribution and levels of various lipids, including cholesterol and glycosylphosphatidylinositol (GPI) anchors. It is involved in the synthesis and proper localization of these lipids, ensuring their availability for vital cellular processes. Additionally, ARV1 is connected to the ER-associated degradation (ERAD) pathway, which recognizes and breaks down misfolded proteins to provide protein quality control [1]. Dysfunction or mutations in the ARV1 gene can lead to severe metabolic disorders and have been linked to conditions such as congenital disorders of glycosylation and epilepsy [1,2]. Through its multifaceted roles, ARV1 contributes significantly to the proper functioning of cellular membranes and overall cell health.

To date, there have been approximately 20 reported cases of ARV1 gene mutations in the literature [3]. ARV1 deficiency clinically manifests as autosomal recessive developmental and epileptic encephalopathy 38 (DEE38) in humans [4]. In the literature, a number of variant mutations in the ARV1 gene have been identified, though they are relatively limited. Patients with ARV1 gene mutation present with several clinical manifestations including developmental milestone delay, intellectual disability, epileptic encephalopathy of infantile onset, hypotonia, ataxia, ophthalmological manifestations (visual impairment, retinal dystrophy), hearing loss, skeletal dysplasia, and dilated cardiomyopathy [4-8]. In this detailed case report, we present three distinct cases of pediatric patients diagnosed with epileptic encephalopathy and developmental delay.

## Case presentation

Case 1

A four-month-old girl from a consanguineous marriage presented to our hospital with multiple episodes of convulsions in the form of generalized body stiffness associated with loss of consciousness, eye up-rolling, and peri-oral cyanosis lasting for two minutes, followed by post-ictal drowsiness. There was no history of fever or vomiting. Her prenatal history was unremarkable, with a negative family history of seizure disorder.

Her initial vital signs were normal. Neurological assessment showed global developmental delay in the form of poor head control and inability to roll over the bed. She had poor social communication as she was not cooing/babbling and did not show any social smile. Muscle tone and power, deep tendon reflexes, and cranial nerves examination were within normal limits with no cerebellar signs. There were no dysmorphic facial features or neurocutaneous skin lesions. The rest of the physical examination was unremarkable.

**Figure 1:**
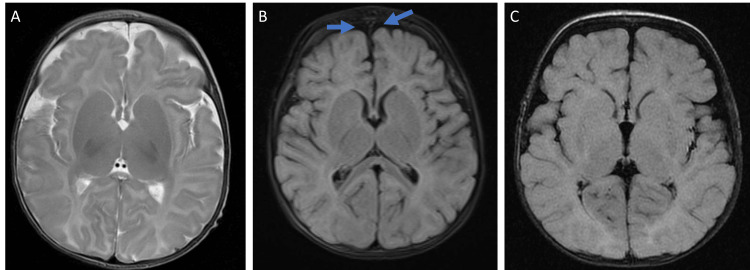
Brain MRI of the three reported ARV1 cases. (A) Unremarkable axial T2-weighted brain MRI. (B) T2-weighted fat-stranding brain MRI showed slightly enlarged bifrontal subarachnoid spaces (arrow). (C) Unremarkable axial FLAIR-weighted brain MRI.

Upon admission, the patient had frequent seizure episodes that were treated with multiple antiepileptic medications, including diazepam, phenobarbital, levetiracetam, vigabatrin, topiramate, lacosamide, and carbamazepine. Her initial brain computed tomography (CT) scan and magnetic resonance imaging (MRI) were unremarkable (Figure 1A). EEG showed modified hypsarrhythmia along with right temporal electrographic seizure activity. The tandem mass spectrometry (TMS) screening showed no indication of conditions associated with amino acid, organic acid, or fatty acid disorders. Additionally, whole-exome sequencing found a homozygous variant of unknown significance in the ARV1 gene (p.Cys61Tyr).

The patient's condition was complicated by episodes of fever and subsequent development of pneumonia, sepsis, acute liver failure, and stage III hepatic encephalopathy. The lab (Table 1) and ultrasound findings indicate a critically ill pediatric patient with multiple organ dysfunction. The complete blood count (CBC) reveals leukocytosis with white blood cells (WBC) of 19 × 10⁹/L (reference range: 5-16 × 10⁹/L) as well as anemia with hemoglobin (Hb) at 11 g/dL (reference range: 10-14 g/dL). The lactate dehydrogenase (LDH) levels were significantly elevated, rising from 2830 U/L to 9865 U/L (reference range: 140-280 U/L) with deranged coagulation profiles consistent with disseminated intravascular coagulation (DIC). Liver function tests (LFTs) demonstrate severely elevated alanine transaminase (ALT) and aspartate transaminase (AST) levels, with total bilirubin rising from 40 umol/l to 60 umol/l (reference range: 0-17 umol/l) and direct bilirubin from 31 umol/l to 47 umol/l (reference range: 0-3.4 umol/l), indicating acute liver injury and cholestasis. Electrolyte levels are mostly within normal limits. Ammonia levels have dramatically increased from 69 to 259 μmol/L (reference range: <50 μmol/L), indicating severe hepatic encephalopathy. Urine and blood cultures were negative. Ultrasound findings revealed signs of hepatitis and significant ascites. These findings are consistent with acute liver failure, with severe coagulopathy and hepatic encephalopathy, requiring immediate and comprehensive medical intervention. Despite extensive management of the patient with broad-spectrum antibiotics, multiple doses of vitamin K, and fresh frozen plasma, the patient’s condition continued to deteriorate the patient eventually expired.

**Table 1:** Laboratory results for ARV1 cases. WBC: white blood cell; LDH: lactate dehydrogenase; ALT: alanine transaminase; AST: aspartate transaminase

Lab Parameter	Normal Range	Case 1	Case 2	Case 3
WBC count (×10⁹/L)	5 – 16	19	Not mentioned	Not mentioned
Hemoglobin (g/dL)	10 – 14	11	Not mentioned	Not mentioned
LDH (U/L)	140 – 280	2830 → 9865	Not mentioned	Not mentioned
ALT / AST (U/L)	ALT: <40 / AST: <40	Severely elevated (no exact values)	Not mentioned	Not mentioned
Total bilirubin (µmol/L)	0 – 17	40 → 60	Not mentioned	Not mentioned
Direct bilirubin (µmol/L)	0 – 3.4	31 → 47	Not mentioned	Not mentioned
Ammonia (µmol/L)	<50	69 → 259	Not mentioned	Not mentioned
Urine/blood cultures	Negative	Negative	Not mentioned	Not mentioned
TMS screening	Negative	Negative	Negative	Negative
Urine organic acids	Negative	Not mentioned	Not mentioned	Negative
Genetic test (ARV1 gene)	–	Homozygous VUS (p.Cys61Tyr)	Homozygous pathogenic (p.Phe144Argfs*5)	Homozygous VUS (p.Cys61Tyr)

Case 2

A female child aged six months and 25 days presented for investigation for global developmental delay. The manifestation started at the age of three months when the mother noticed that her baby did not fixate or follow with an upward gaze, did not support her head, and was floppy without any history of abnormal movement, as well as seizure-like activity, choking, or feeding difficulties. The baby was born from a consanguineous marriage and had a four-year-old healthy brother. No family history of epilepsy or special needs child. The baby had a history of hospital admission at the age of four months due to bronchiolitis. The baby was up-to-date on her vaccinations and was on formula feeds and soft, solid food. She was born at 37 weeks with a birth weight of 3 kg without any history of perinatal complications or neonatal intensive care unit (NICU) admission.

The baby’s developmental milestones showed the following: poor head control, does not sit with support, does not roll over from supine to prone position, can grasp/hold objects briefly, does not transfer, and can coo but does not babble.

Physical exam showed an active child, an anterior fontanelle at level, normal power and tone, normal reflexes, upward gazing, does not fix or follow, head lag on pulling, horizontal and vertical nystagmus with facial dysmorphic features in the form of macrocephaly, short neck, and low-set ears. The rest of the systemic examination was unremarkable.

The patient also developed generalized tonic-clonic seizures of both upper and lower limbs associated with drooling, upward deviation of eyes, with loss of consciousness, which lasted for forty minutes, which was managed by diazepam and phenytoin, and then kept on maintenance keppra. Brain MRI showed slightly enlarged bifrontal subarachnoid spaces with an abnormal finding noticed in the right parietal lobe (Figure 1B). The tandem mass spectrometry (TMS) screening showed no indication of conditions associated with amino acid, organic acid, or fatty acid disorders.

The whole-exome sequencing revealed a homozygous pathogenic variant in the ARV1 gene (p.Phe144Argfs*5). The genetic diagnosis of autosomal recessive developmental and epileptic encephalopathy-38 was confirmed. The patient remained stable in the hospital with no further attacks and seizures, was discharged home in good condition with arrangements for genetic counselling to the parents.

Case 3

A three-month-old girl, born at 35 weeks of gestation, a known case of gastroesophageal reflux disease (GERD) on anti-regurgitation (AR) formula and domperidone (1mg QID), presented to the emergency department with a history of abnormal movement that started one day before admission. These abnormal movements occurred three times the day before admission and twice on the day of admission. The episodes were described as up-rolling of the eyes with lip smacking or shivering. Episodes were followed by drowsiness and a post-ictal phase. No history of cyanosis, stiffness, convulsions, loss of consciousness, fever, cough, or shortness of breath. The baby was feeding well with no vomiting. The mother reported a history of colic and passing motion every two days with straining. The baby’s developmental history included head lag and poor head control, not yet smiling, with a startle response present. The patient was born through cesarean delivery with a history of NICU admission due to respiratory distress syndrome (RDS). The birth weight was 2.470 Kg and the weight at admission was 3.9 (below 3rd centile). Family history was significant for epilepsy in a 19-year-old cousin.

The patient's vitals were stable. She looked well, pink, active, not in distress, and was well-hydrated with a capillary refill time of less than 2 seconds. Dysmorphic features were noticed and included a low hairline, micrognathia, hypertelorism, and a depressed nasal bridge. The rest of the systemic examination was unremarkable. Neurological exam revealed head lag, truncal hypotonia, brisk reflexes of +3 with no clonus, intact cranial nerve examination, normal anterior fontanelle, and positive Moro reflex.

**Figure 2:**
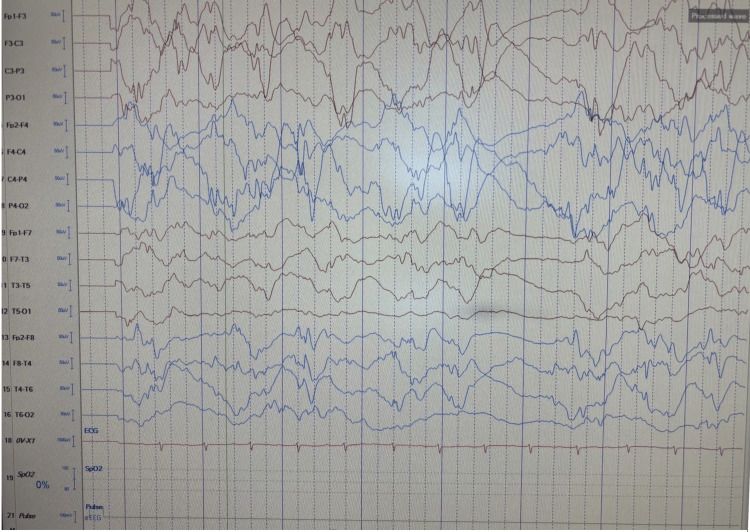
Brain EEG showed a presence of multi-focal epileptiform spike and wave discharges.

Brain MRI was unremarkable (Figure 1C). Ophthalmology evaluation revealed normal fundoscopic examination, however, EEG showed the presence of multifocal epileptiform spike and wave discharges (Figure 2). Urine organic acid screening was performed and showed no indication of organic disease. The tandem mass spectrometry (TMS) screening showed no indication of conditions associated with amino acid, organic acid, or fatty acid disorders. In addition, whole-exome sequencing found a homozygous variant of unknown significance in the ARV1 gene (p.Cys61Tyr).

The patient also developed an episode of seizure activity in the form of eye blinking with lip smacking, followed by a post-ictal phase. Seizures were controlled by levetiracetam. As a result, the patient was discharged on levetiracetam with follow-ups in the clinic for genetic study. The patient was admitted multiple times due to seizure breakthrough with presumed sepsis due to gastroenteritis or pneumonia. The latest discharge medications included diazepam, topiramate, levetiracetam, and phenytoin as antiepileptic medications, with genetic counselling for the parents.

The following table summarizes the three reported cases (Table 2).

**Table 2:** Summary of significant findings in the described cases. EEG: electroencephalogram; CT: computed tomography; MRI: magnetic resonance imaging; TMS: transcranial magnetic stimulation.

Aspect	Case 1	Case 2	Case 3
Age of onset	2 years 11 months	6 months	3 months
Gender	Female	Female	Female
Consanguineous marriage	Present	Present	Present
Symptoms	Recurrent seizures, developmental delay, and dysmorphic features	Significant global developmental delay, dysmorphic features, and seizure	Seizure, developmental delay, and dysmorphic features
Key diagnostics	Normal EEG, brain CT/MRI normal, TMS screening negative.	Normal EEG, MRI with parietal abnormality, TMS screening negative.	Multi-focal epileptiform discharges on EEG, brain MRI, slightly enlarged bifrontal subarachnoid spaces with abnormal findings noticed in the right parietal lobe, TMS screening negative.
Genetic findings	ARV1 mutation (p.Cys61Tyr).	ARV1 mutation (p.Phe144Argfs*5), confirmed DEE-38.	ARV1 mutation (p.Cys61Tyr).
Outcome	Death	Alive	Alive

## Discussion

The ARV1 gene plays a critical role in lipid metabolism, thus maintaining cellular homeostasis [4]. ARV1 is essential for the regulation and distribution of sterols and sphingolipids within the cell, which are responsible for cell membrane integrity and function [4]. Additionally, ARV1 is involved in the synthesis of glycosylphosphatidylinositol (GPI) anchors, which function as attaching proteins to cell membranes [4]. Therefore, dysfunctions in ARV1 can lead to membrane disorganization, hypersensitivity to fatty acids, and endoplasmic reticulum stress. Impairment in sphingolipid metabolism has been identified to be involved in many neurodegenerative diseases, such as Niemann-Pick disease [5]. ARV1 gene mutation in humans and its association with neurological diseases was first described in 2015 by Alazami et al. after conducting whole-exome sequencing on 143 multiplex families with a diagnosis of neurogenetic disease, born from consanguineous marriage with positive family history [5]. 

This case series discusses three cases of infants from consanguineous marriages who exhibited recurrent breakthrough seizures, severe developmental delay, and dysmorphological features. These infants were found to possess ARV1 gene mutations through whole-exome sequencing, a known genetic cause of developmental and epileptic encephalopathy.

The first ARV1 gene variant mutation that was identified in two of our patients was p.Cys61Tyr. This variant mutation is an amino acid change from cysteine to tyrosine at position 61. This variant mutation had been identified by Salian et al. in an 11-year-old male who presented at the age of 6 months with developmental delay, profound intellectual disability, and seizure with a history of nephrocalcinosis [9]. The patient had a normal prenatal and neonatal history without any history of congenital microcephaly, feeding difficulties, or failure to thrive. Additionally, no facial dysmorphic features were observed. Ophthalmology evaluation revealed abnormal vision and strabismus. Finally, skeletal examination and survey identified kyphosis, pectus excavatum, and hip subluxation. In the present study, two patients were identified to have p.Cys61Tyr variant mutation. Both patients presented with developmental delay, dysmorphic features, and seizures.

The second ARV1 gene mutation that was identified was p.Phe144Argfs*5. This variant mutation involves a frameshift alteration starting at phenylalanine (Phe) at position 144, resulting in a change in the reading frame and the introduction of a premature stop codon five amino acids. To the best of our knowledge, this variant has not been previously documented in the literature. It has been classified as pathogenic according to the implementation of the ACMG/AMP/ClinGen SVI guidelines by CENTOGENE.

Palmer et al/ identified a variant AVR1 mutation referred to as p.(Lys59_Asn98del) in a 4-month-old female patient who presented with intractable seizure [4]. This mutation indicated that there was a loss of amino acids from lysine at position 59 to asparagine at position 98. Furthermore, this mutation was evaluated for protein expression and function by adding the mutated AVR1 gene in yeast lacking the ARV1 gene. This variant did not restore the yeast's ability to grow at high ("restrictive") temperatures, suggesting that this deletion severely impairs the function of the ARV1 protein. Davids et al. described homozygous splice-variant ARV1 gene mutations in seven patients who presented with seizure of infantile onset, profound developmental delays, substantial hypotonia, speech delay, visual impairment, and severe generalized brain atrophy [6]. Using immunofluorescence microscopy and the fluorescence-activated cell sorting (FACS) analysis, the effect of these variant mutations on the expression of GPI-anchored proteins was analyzed and demonstrated reduced expression of these proteins at the surface of fibroblasts and neutrophils [5]. Segel et al. concluded that the p.Gly189Arg mutation in the ARV1 gene is associated with a defect in GPI anchoring function, which consequently results in infantile epileptic encephalopathy in two brothers [7]. Interestingly, the two brothers were reported to have cardiac involvement presented as dilated cardiomyopathy. Cardiac involvement in patients with ARV1 gene mutations was additionally documented in two recent studies [9,10].

Salian et al. reported five novel mutation variants in the ARV1 gene in a cohort of seven patients with developmental delay, refractory seizures of early onset, hypotonia, with specific features on neuroimaging [9]. The variant mutations identified were as follows: p.(Ser122Glnfs7) in one patient, a compound heterozygous mutation of p.(Pro174Alafs14) and p.(Cys34Tyr) in another patient, p.(Cys61Tyr) in a different patient, c.674-1G > A in two patients, and p.(Pro174Alafs*14) in yet another patient [9]. They also noted that patients with ARV1 mutations exhibit a range of clinical manifestations other than the typically observed severe epileptic encephalopathy. These include variable skeletal abnormalities, genitourinary issues, nephrocalcinosis, uterine and renal congenital anomalies, and abnormal cardiac morphology [9].

Darra et al. described two sisters from a non-consanguineous marriage who presented with dyskinetic movements, visual inattention, hypotonia, and profound developmental delay accompanied by abnormally slow EEG activity. These symptoms were associated with compound heterozygous AVR1 gene mutations, specifically p.Ser122Glnfs*7 and the p.Trp163* variants [8]. The most recent AVR1 gene mutation (p.L185del) was identified by Karabinos et al. in an adult male patient who presented with intellectual disability, developmental delay, intractable seizures, difficulties in speech and movement, and dilated cardiomyopathy [10].

## Conclusions

In this study, we presented three cases demonstrating the significant and varied impact of ARV1 gene mutations on pediatric patients. Profound developmental delays, intractable seizures, and dysmorphic features were the presenting symptoms in all cases. Identification of the ARV1 mutation in all of the cases suggests the critical role of this gene in neurological development and function. Therefore, this case series contributes to further understanding of AVR1-associated neurological disorders and highlights the importance of genetic testing in the diagnosis and management of similar conditions. Looking forward, future researchers into ARV1 may uncover its further roles in neurodevelopment, guide the development of targeted diagnostic tools, and potentially open new plans for therapeutic interventions aimed at modifying disease progression in affected individuals.
